# Knowledge, attitude, and practice pattern towards diabetic retinopathy screening among general practitioners in primary health centres in Jakarta, the capital of Indonesia

**DOI:** 10.1186/s12875-023-02068-8

**Published:** 2023-05-11

**Authors:** Yeni Dwi Lestari, Gitalisa Andayani Adriono, Rizka Ratmilia, Christy Magdalena, Ratna Sitompul

**Affiliations:** Ophthalmology Department, Faculty of Medicine, Universitas Indonesia-Cipto Mangunkusumo General Hospital, Jakarta, Indonesia

**Keywords:** KAP, Diabetic retinopathy, Primary health centre, General practitioner

## Abstract

**Background:**

Diabetic retinopathy (DR) is an emerging cause of visual impairment and blindness and is often detected in the irreversible stage. General practitioners (GPs) play an essential role in the prevention of DR through diabetes control, early detection of retinal changes, and timely referral to ophthalmologists. This study aimed to determine the knowledge, attitude, and practice (KAP) towards DR screening among GPs in the district primary health centres (PHCs) in Jakarta, Indonesia.

**Methods:**

A cross-sectional study was conducted between April 2021 and February 2022 in 17 randomly selected district PHCs. A validated online questionnaire was then distributed. Good knowledge was defined when the correct response rate was > 75%, positive attitude was indicated when desired attitudes were found in more than half of the items (> 50%), and good practice was defined when more than half of the practice items (> 50%) were performed.

**Results:**

A total of 92 GPs, with a response rate of 60.1%, completed the questionnaire. Seventy-nine respondents (85.9%) were female with a median (range) age of 32 (24–58) years. Among the respondents, 82 (89.1%) had good knowledge and all showed positive attitude on DR screening. However, only four (4.3%) demonstrated good practices. We found a weak positive correlation (r_s_ = 0.298, p = 0.004) between attitude and practices.

**Conclusion:**

GPs in Jakarta showed good knowledge and positive attitude on DR screening. However, they did not show good practice. There was a positive correlation between attitude and practice.

**Supplementary Information:**

The online version contains supplementary material available at 10.1186/s12875-023-02068-8.

## Introduction

Diabetes mellitus (DM) is a major public health problem in Indonesia. [1] According to the International Diabetes Federation, Indonesia ranks fifth globally in terms of the highest number of people with diabetes (PwD) and this number will increase from 19.5 million to 28.6 million in 2045. [2].

Diabetic retinopathy (DR) is one of the leading causes of visual impairment and blindness among the working age population globally. [3] As the global prevalence of diabetes increases, the prevalence of DR and vision-threatening diabetic retinopathy (VTDR) are also estimated to increase. [4] A population-based study reported that the prevalence of DR and VTDR were 43.1% and 26.3%, respectively, among Indonesian adults with type 2 DM in the urban and rural areas. [5] Approximately one in four adults with diabetes had VTDR, and 1 in 12 of those with VTDR was bilaterally blind, suggesting the need for effective screening and management of DR. [5].

Timely detection and management of DR could prevent more than 90% of diabetes-related vision loss. [6] The asymptomatic nature of DR and absence of DR screening practice may lead to delays in diagnosis and management. [7] Sasongko et al. reported that 94.9% of PwD in a rural area in Yogyakarta, Indonesia, had not undergone eye examination. [8] Another study showed that a low referral rate accounts for late presentation to ophthalmologists. [9] This underscores the importance of implementing DR screening at the primary care level. In Indonesia, general practitioners (GPs) are the only health professionals who are competent in performing fundus examinations at the primary care level.

In Jakarta, 84.7% of PwD had not undergone eye examination in the past year, and less than half (49.4%) of all patients were told of the need for it. [10] This finding is a major concern because Jakarta is the capital city of Indonesia, with an urban population that continues to increase every year along with increasing economic growth. In Indonesia, primary health centres (PHCs) are government-mandated health clinics at the primary care level. By 2020, there were 340 PHCs in Jakarta, consisting of 44 district health centres and 296 subdistrict health centres. [11] A few services are available at PHCs that provide care for PwD, namely, non-communicable disease clinics, elderly health clinics, and general clinics. [11].

Several studies assessed GPs’ knowledge, attitude, and practice (KAP) towards DR in other countries. [12–15] However, data of KAP towards DR screening among GPs in Indonesia are limited, with only one study conducted in Bandung. [16] This study aimed to determine the KAP level towards DR screening among GPs as well as barriers that may hinder screening at PHCs in Jakarta.

## Materials and methods

### Study design and study population

This cross-sectional study was conducted from April 2021 to February 2022 alongside our main research project, which studied DR prevalence in Jakarta by collecting data from 17 district PHCs. Seventeen of 44 district PHCs were selected based on proportional multistage random sampling in each administrative city in Jakarta. District PHCs were preferred because diabetes services are mainly conducted in this area. We distributed a questionnaire through one person-in-charge at each PHC to all 153 GPs in these PHCs.

The sample size for correlation study was calculated with a 95% confidence level, 5% type I and type II errors. Expected correlation was set at 0.5. [16] The final minimum sample size was 46. We included all GPs who worked at the selected PHCs and agreed to complete the questionnaire. The exclusion criterion for this study was incomplete responses.

### Questionnaire

The online questionnaire used in this study was adapted from a previous study that had been validated for the Indonesian population (Supplementary File 1). [16] The KAP questionnaire comprised closed-ended questions consisting of four sections: demographic characteristics, DR-related knowledge, attitude of GPs towards DR screening, and evaluation of practice and barriers in performing DR screening. The last section was an open-ended question that allowed respondents to list the perceived barriers to DR screening.

### Scoring

The knowledge section of the questionnaire consisted of 36 questions, of which 10 questions allowed multiple answers. One point was awarded for a correct response, and 0 points were given for incorrect responses. The maximum score on the knowledge section was 36. The sum of all answers to knowledge-related questions was further graded as follows: good, if the score was > 75% or > 27 correct responses; fair, if the score was between 50% and 75% or 18–27 correct responses; and poor, if the score was < 50% or < 18 correct responses.

The attitude section comprised eight questions using the 5-point Likert scale, which had five possible responses: strongly agree, agree, uncertain, disagree, and strongly disagree. Five points were awarded for the most favourable response, and one point was given for the least favourable response. The maximum score for the attitude section was 40. Attitude was considered positive if the score was ≥ 50% or there were at least 20 favourable responses, and a score of < 50% or < 20 favourable responses were categorised as negative attitude.

The practice section of the questionnaire consisted of six questions, with one point awarded for each item that was performed. Practices were categorised as good practice if the score was ≥ 50% or more than three practice items were performed and poor practice if the score was < 50% or less than three practice items were performed. The maximum score for the practice section was 6. The summary of the scoring system for KAP is presented in Table 1.

**Table 1:** Scoring system for KAP questionnaire

Domain	Categories	Correct/favourable responses
Knowledge	Good	> 75% correct responses
	Poor	< 75% correct responses
Attitude	Positive	$\ge$50% or at least 20 favourable responses
	Negative	< 50% or < 20 favourable responses
Practice	Good	$\ge$50% or more than 3 items were performed
	Poor	< 50% or < 3 items were performed

For perceived barriers, we pooled all barriers, grouped them under similar categories, and calculated the frequency of each category. The barriers categories are as follows: “lack of facilities”, “knowledge- and skill-related factors”, “patient-related barriers”, “time constraint”, “lack of human resources”, “lack of fundoscopy training”, “referral limitation”, and “believe it was not part of primary health care”.

### Statistical analysis

Data were coded and recorded on a spreadsheet. Statistical analysis was performed using the Statistical Package for the Social Sciences version 25.0. Categorical variables are presented as frequencies and percentages.

This study also conducted a correlation analysis to determine the relationship between the KAP. Furthermore, we performed a Kurtosis and Skewness analysis followed by the Kolmogorov–Smirnov test to assess the normality assumption of the collected data. The Kolmogorov–Smirnov test demonstrated the following results: knowledge, p = 0.000 (p < 0.05); attitude, p = 0.200 (p > 0.05); and practice, p = 0.000 (p < 0.05). Of the three variables, only “attitude” was normally distributed. Therefore, we performed the non-parametric Spearman correlation test to identify the correlation between knowledge and attitude, knowledge and practice, and attitude and practice. A p-value of < 0.05 identified through two-tailed tests was considered to be statistically significant. The Guilford criteria were used for the strength of the following: 0.0 to < 0.2, very weak; 0.2 to < 0.4, weak; 0.4 to < 0.7, moderate; 0.7 to < 0.9, strong; and 0.9–1.0, very strong. [17] A p-value of < 0.05 meant that there was a significant correlation between the two variables tested.

### Ethical consideration

This study adhered to the guidelines of the Declaration of Helsinki, and ethical clearance was obtained from the Universitas Indonesia Ethical Review Board (KET-1418/UN.2F1/ETIK/PPM.00.02/2020) on 30 November 2020. Informed consent was obtained on the first page by answering a yes-or-no question before proceeding to the next page containing the survey.

## Results

### Demographic characteristics

A total of 92/153 GPs completed the questionnaire, yielding a response rate of 60.1%. Most of them were females (79/92, 85.9%), with a median (range) age of 32 (24–58) years. More than half of the respondents (50/92, 53.8%) were between the ages of 30 and 35 years, and 52/92 (56.5%) had 1–5 years of experience in practice, as shown in Table 2.

**Table 2:** Sociodemographic characteristics of study respondents (N = 92)

Characteristics	n
Age, median (range), year	32 (24–58)
20–29	28 (30.4)
30–39	50 (53.8)
40–49	9 (9.8)
≥ 50	5 (5.4)
Sex, No. (%)	
Male	13 (14.1)
Female	79 (85.9)
Duration of practice, No. (%)	
0–5 years	52 (56.5)
6–10 years	23 (25)
> 10 years	17 (18.5)
Region of PHCs, No. (%)	
Central Jakarta	31 (33.7)
South Jakarta	18 (19.6)
West Jakarta	4 (4.3)
North Jakarta	8 (8.7)
East Jakarta	31 (33.7)

PHCs, primary health centres

An overview of the KAP scores is presented in Table 3. This study indicates that 82/92 (89.1%) of GPs in Jakarta had a good level of knowledge and positive attitude. However, only 4/92 (4.3%) were classified as having a good level of DR screening practice.

**Table 3:** Overview of the knowledge, attitude, and practice scores

Variables	Frequency (n)	Percentage (%)	
Knowledge (median [range]) 30.0 [21–35)			
Good	82	89.1	
Fair	10	10.9	
Poor	0	0	
Attitude (median [range]) 32.0 [23–37]			
Positive	92	100	
Negative	0	0	
Practice (median [range]) 1.0 [0–4)			
Good	4	4.3	
Poor	88	95.7	

Table 4 shows the distribution of answers to each item assessing knowledge. Most participants had poor knowledge about DR detection in type 1 DM (T1DM) with only (14/92, 15.0%) correct responses. Correct responses were the highest in items about the prevention of DR complications (87/92, 94.6%).

**Table 4:** Knowledge regarding DR

Knowledge question	Response (n = 92)	
	**Right**, **n (%)**	**Wrong**, **n (%)**
DM vascular complications	63 (68.0)	29 (32.0)
Factors that aggravated DR	64 (70.0)	28 (30.0)
Recommended eye examination in type 2 DM	63 (68.0)	29 (32.0)
Recommended eye examination in type 1 DM	14 (15.0)	78 (85.0)
Recommended eye examination in patients with diabetes with pregnancy	52 (56.0)	40 (44.0)
Symptoms of DR in patients with diabetes	62 (67.0)	30 (33.0)
Retinal changes observed in direct fundoscopy	57 (62.0)	35 (38.0)
Timely fundoscopic examination of patients with diabetes without DR	51 (55.0)	41 (45.0)
Prevention of DR	87 (94.6)	5 (5.4)
Management of DR	63 (68.5)	29 (31.5)

DM, diabetes mellitus; DR, diabetic retinopathy

The responses to statements about attitudes towards performing eye examinations, referral to an ophthalmologist, and early detection of DR in PwD are presented in Table 5. The majority of GPs agreed that all PwD should undergo regular eye examinations despite good glycaemic control (88/92, 95.7%) and should be referred to ophthalmologists (75/92, 81.5%). Most GPs disagreed that eye examination should be performed only in the presence of symptoms (80/92, 87.0%) and that direct fundoscopic examination using direct ophthalmoscopy should be performed by an ophthalmologist only (56/92, 60.8%). Most respondents (90/92, 97.9%) agreed that blindness due to DR could be prevented with early treatment of diabetes and that non-ophthalmologists could help detect DR (74/92, 80.5%). Most GPs (60/92, 65.2%) felt that they were adequately trained in managing patients with eye complaints.

**Table 5:** Attitude towards DR screening

Attitude questions	Strongly disagree, n (%)	Disagree, n (%)	Don’t know, n (%)	Agree, n (%)	Strongly agree, n (%)
Eye examination is only needed when vision is affected	16 (17.4)	64 (69.6)	1 (1.1)	11 (12)	0 (0.0)
All patients with diabetes should be referred to an ophthalmologist	1 (1.1)	13 (14.1)	3 (3.3)	36 (39.1)	39 (42.4)
Even though diabetes is controlled, patients still have to undergo routine eye examinations	0 (0.0)	1 (1.1)	3 (3.3)	34 (37)	54 (58.7)
If the doctor has told the patient with diabetes to come for routine follow-up, the patient will come	0 (0.0)	2 (2.2)	7 (7.6)	24 (26.1)	59 (64.1)
If diabetes is treated early, blindness due to DR can be prevented	1 (1.1)	0 (0)	1 (1.1)	11 (12.0)	79 (85.9)
Fundoscopic examination should be performed by an ophthalmologist only	5 (5.4)	51 (55.4)	7 (7.6)	20 (21.7)	9 (9.8)
Fundoscopic examination by a non-ophthalmologist can help detect diabetic retinopathy	2 (2.2)	7 (7.6)	9 (9.8)	41 (44.6)	33 (35.9)
Ophthalmology training in a medical school adequately equips the GP to manage patients with eye complaints	0 (0.0)	27 (29.3)	5 (5.4)	40 (43.5)	20 (21.7)

DR, diabetic retinopathy; GP, general practitioner

Fifty-two (52/92, 56.5%) GPs conducted visual acuity examination, and twelve (12/92, 13%) GPs had access to an ophthalmoscope at their workplaces. However, fundus examination was performed by only 3/92 (3.3%) GPs in PwD. Thirteen (13/92, 14.1%) GPs had attempted to perform fundus examination in the past 6 months. Sixty-five GPs (65/92, 70.7%) referred PwD to ophthalmologists for eye examinations. Only ten (10/92, 10.9%) GPs attended seminars or training on DM and DR in the past year, as shown in Table 6.

**Table 6:** DR screening practice

Practice questions	Yes, n (%)	No, n (%)
Do you test the vision of your patient with diabetes	52 (56.5)	40 (43.5)
Do you examine the fundus (retina) of your patient with diabetes	3 (3.3)	89 (96.7)
Do you refer patients with diabetes for eye examination	65 (70.7)	27 (29.3)
Do you always have access to an ophthalmoscope at your workplace	12 (13)	80 (87)
Have you ever tried to perform fundus examination on your patient with diabetes for the past 6 months	13 (14.1)	79 (85.9)
Did you attend any seminar/training about DM and DR in the past year	10 (10.9)	82 (89.1)

DM, diabetes mellitus; DR, diabetic retinopathy

**Fig. 1:**
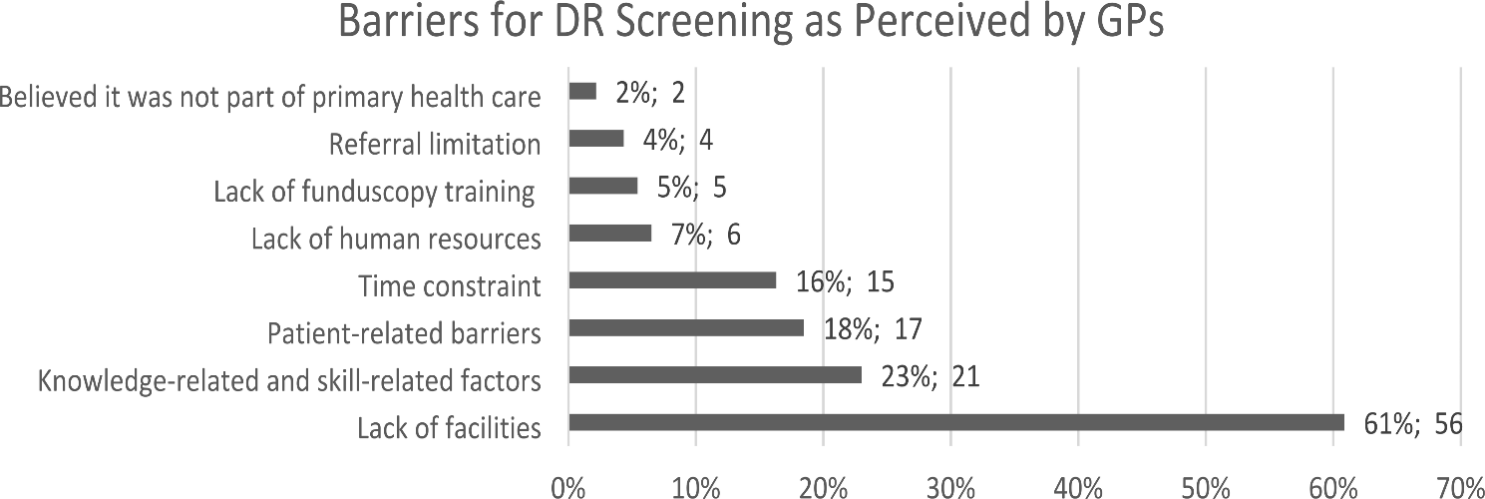
Perceived barriers to diabetic retinopathy screening

The perceived barriers to DR screening are shown in Fig. 1. Lack of facilities (56/92, 61%), such as ophthalmoscopes, mydriatic eye drops, proper rooms for eye examinations, and portable fundus cameras, was the main barrier, followed by knowledge and skill-related factors (21/92, 23%). Furthermore, (17/92, 18%) GPs perceived 18% of the barriers to be patient-related, such as unawareness of the asymptomatic nature of DR, lack of compliance, lack of understanding of annual DR screening, lack of assistance, and poor family support.

The correlations between the KAP were evaluated using the Spearman correlation analysis (Table 7). There was a significant yet weak positive correlation between attitude and practice (r_s_ = 0.298, p = 0.004).

**Table 7:** Correlation between the knowledge, attitude, and practice of a general practitioner to diabetic retinopathy screening

Variables	Correlation coefficient	p-value
Knowledge and attitude	−0.008	0.943
Knowledge and practice	0.007	0.950
Attitude and practice	0.298	0.004*

Sig (two-tailed) * marks indicate a very highly significant correlation, p < 0.05.

## Discussion

This cross-sectional study examined the levels and determinants of KAP towards DR screening among GPs at district PHCs, which are government-mandated health clinics in Jakarta. We recruited respondents during our main research project that studied DR prevalence in Jakarta. Our findings indicate that a large percentage of GP had good knowledge of DR, positive attitude, but poor practices.

This study revealed good knowledge regarding DR screening among GPs. Almost all GPs (94.6%) knew about the prevention of DR, similar to the findings reported by Edwiza et al. [16] in Bandung, Indonesia (97.5%), and Al Ghamdi et al. [12] in Taif, Saudi Arabia (92.8%). Since 2020, DM management and its complications have been a priority in Jakarta. Hence, many webinars and training opportunities have been held to increase the awareness of DM and DR among GPs before our data collection.

One most incorrectly answered question was about the recommended initial DR screening following T1DM diagnosis. This is in line with the findings of a study conducted in Tabuk, Saudi Arabia. [13] This discrepancy suggests that there was a lower level of comprehension of T1DM screening recommendation, which may be associated with their lower prevalence in Indonesia and the fact that most patients with T1DM are under direct care by endocrinologists in tertiary healthcare. [13].

All GPs demonstrated positive attitudes towards DR screening. The majority (87%) disagreed that eye examination is only needed when vision is affected, and 80.5% agreed that fundoscopic examination by non-ophthalmologists can help reduce vision problems due to diabetes. The studies conducted in Sudan and Nepal have reported similar findings. [14, 15]

Despite their good level of knowledge and attitude, GPs showed poor practice in DR screening. This discrepancy was also reported in previous studies. [12] Poor practice was related to fundus examination and ophthalmoscope access, which are in alignment with studies in Sudan and Bandung, Indonesia. Although a direct ophthalmoscope is a standard instrument in PHCs, it is still not widely available, thus hindering GPs from performing their duties. Nevertheless, similar to the findings in a previous study, GPs tend to refer patients to ophthalmologists for examination. [14, 16] A qualitative study highlighted that GPs faced role confusions in DR screening as some GPs considered themselves as referral source. [6] If every individual with diabetes is referred to ophthalmologists for examination, this will increase the burden on ophthalmologists and the healthcare system. Consequently, patients may be discouraged from visiting ophthalmologists for reasons because of the high number of patients in secondary and tertiary hospitals, fees incurred for travel, and the distant feeling being cared by doctors other than their GPs. Therefore, referral adherence still varies, ranging from 29.9–91.9%.[18–20]

Barriers to DR screening vary according to the country’s income level and various system factors in each setting. Different economic and socio-cultural factors would affect the implementation. [21] Management of DR in low-middle income countries faces major challenges due to lack of access to ophthalmologists, healthcare resources, and facilities. [22] The main barrier reported in this study was lack of facilities, such as ophthalmoscopes, mydriatic eye drops, and proper rooms for eye examinations.

GPs’ lack of experience and confidence in performing fundus examinations and diagnosing fundus abnormalities have also been highlighted in other studies. [6, 12, 15] This is coupled with the fact that only 10.9% of respondents had training in eye examinations in patients with diabetes in the past year. PHCs and GPs should be equipped to detect DR as it is a part of GPs’ competence, and the spirit of continuing medical education should be encouraged.

In urban areas, such as Jakarta, implementing a DR screening model using a fundus camera integrated with telemedicine and automated grading by artificial intelligence (AI) is a promising solution. Currently, fundus cameras are the standard equipment, and automated grading by AI has demonstrated good diagnostic accuracy for screening. With this model, DR screening coverage may improve.

Our study found that a significant percentage of respondents referred patients for ophthalmic examination and admitted that they did not try to perform it themselves. A few respondents believed that GPs’ roles did not include DR screening. A study in Australia reported that GPs assumed that ophthalmologists were more suited to perform the task. [6] Efforts should be made to clarify the roles of GPs in diabetes management, including screening for possible complications without delaying referral to ophthalmologists once it is appropriate. Evidence suggests that one of the strategies is a shift in focus on upstream primary approaches targeted at population and community, by promoting a healthier lifestyle and self-management of diabetes and providing equal and wider access to DR screening. [23].

In this study, GPs perceived 18% of the barriers to be patient-related. These included reluctance to referral, low levels of compliance, and socioeconomic problems. If GPs are able to counsel patients and persuade them to cooperate in undergoing annual DR screening and timely management of DR, these issues may be diminished. [24].

We found a positive correlation between attitude and practice of GPs regarding DR screening, which is in contrast to a study reported from a similar setting in Bandung, Indonesia, that showed no significant correlation between attitude and GPs’ practice. [16] In parallel with the study in Taif, Saudi Arabia, no correlation was found between knowledge and practice. [12] Whether existing barriers had interfered with the implementation of knowledge of our respondents is beyond the scope of this study.

One of the limitations of this study is that we were unable to guarantee honest responses, which may have impacted the results.

This study adds to the currently limited body of research on KAP towards DR screening among GPs in Indonesia. Good practices of DR screening among GPs, even in Jakarta, the capital of Indonesia, still need to be developed. The procurement of ophthalmoscopes or other screening modalities in PHCs and training opportunities are recommended to improve the capacity of GPs in DR screening.

## Conclusion

Despite the favourable levels of knowledge and attitude, practice towards DR screening is still poor. Infrastructure and human resources are major factors that should be improved so that DR screening could be part of the management of patients with diabetes in PHCs.

## Electronic supplementary material

Below is the link to the electronic supplementary material.

Supplementary Material 1

## Data Availability

The datasets used and analysed during this study are available upon reasonable request by emailing the corresponding author.

## List of abbreviations

AI: Artificial Intelligence
DM: Diabetes Mellitus
DR: Diabetic Retinopathy
GP: General Practitioner
KAP: Knowledge, Attitude, Practice
PHC: Primary Health Centre(s)
VTDR: Vision-threatening Diabetic Retinopathy
