# Supplementary material for: Knowledge, attitude, and practice pattern towards diabetic retinopathy screening among general practitioners in primary health centres in Jakarta, the capital of Indonesia
Source: BMC Prim Care. 2023 May 11;24:114. doi: 10.1186/s12875-023-02068-8 (PMC10176940; doi:10.1186/s12875-023-02068-8)
Supplement: Supplementary file 1 — Supplementary Material 1 [file 12875_2023_2068_MOESM1_ESM.docx]

**QUESTIONNAIRE OF KNOWLEDGE, ATTITUDE, AND PRACTICE FOR GENERAL PRACTITIONER REGARDING DIABETIC RETINOPATHY**

**Section 1 Demographic**

Filling date :

Age :

Sex : (a) Male (b) Female

Email :

Phone :

Workplace :

Graduation years :

Length of works : (a) 1 – 5 years (b) 6 – 10 years (c) >10 years

**Section 2 – Knowledge**

1. Vascular complications of Diabetes Mellitus can affect organs :

- Brain (a) Yes (b) No
- Kidney (a) Yes (b) No
- Eye (a) Yes (b) No
- Peripheral nerves (a) Yes (b) No
- Foot (a) Yes (b) No
- Stomach (a) Yes (b) No
- Heart (a) Yes (b) No

1. What is factors that aggravating diabetic retinopathy (DR) ?

- Duration of diabetes (a) Yes (b) No
- Lipid profile (a) Yes (b) No
- Smoking (a) Yes (b) No
- Glicemic control (a) Yes (b) No
- Hypertension (a) Yes (b) No
- Alcoholism (a) Yes (b) No
- Obesity (a) Yes (b) No

1. When is the right time for type 2 DM patient to have their eyes checked by an ophthalmologist?
2. Immediately after diagnosed
3. 1 year after diagnosed
4. 2 years after diagnosed
5. When is the right time for type 1 DM patient to have their eyes checked by an ophthalmogist?
6. Immediately after diagnosed
7. 1-2 years after diagnosed
8. 3-5 years after diagnosed
9. When is the right time for gestational diabetic patient to have their eyes checked by an ophthalmogist?
10. First trimester
11. Second trimester
12. Third trimester
13. What is symptomps of DR in diabetic patients ?
14. No symptomp
15. Slow blurry vision and painless
16. Sudden blurry vision and painless
17. Slow blurry vision and pain
18. What are the retinal changes that observed in fundoscopy as a result of DM ?

- Microaneurysm (a) Yes (b) No
- Venous beading (a) Yes (b) No
- Macular degeneration (a) Yes (b) No
- Retinal hemorrhage (a) Yes (b) No
- Retinal detachment (a) Yes (b) No
- Vitreous hemorrhage (a) Yes (b) No
- Neovascularization (a) Yes (b) No

1. If there are no DR symptomps, when should diabetic patients have regular check-ups for funduscopic examination?
2. Every 6 months
3. Every 1 year
4. Every 2 year
5. No need
6. What are the prevention of DR complications in diabetic patients?

- Early diagnosis and referral to an ophthalmologist (a) Yes (b) No
- Controlling serum lipid levels (a) Yes (b) No
- Avoid smoking (a) Yes (b) No
- Controlling blood glucose (a) Yes (b) No
- Controlling blood pressure (a) Yes (b) No
- Controlling body weight (a) Yes (b) No

1. What are the management for DR ?

- Laser photocoagulation (a) Yes (b) No
- Ocular surgery (a) Yes (b) No
- Normalize blood pressure (a) Yes (b) No
- Normalize blood glucose (a) Yes (b) No

**Section 3 – Attitude**

1. Eye examination only needed when vision is affected.

1) Strongly disagree 2) Disagree 3) Don’t know 4) Agree 5) Stongly agree

1. All diabetic patients must be referred to ophthalmologist.

1) Stongly disagree 2) Disagree 3) Don’t know 4) Agree 5) Strongly agree

1. Even though diabetes is controlled, patients still have to do routine eye examinations.

1) Stongly disagree 2) Disagree 3) Don’t know 4) Agree 5) Strongly agree

1. If the doctor has told the diabetic patient to come for routine follow-up, the patient will come.

1) Stongly disagree 2) Disagree 3) Don’t know 4) Agree 5) Strongly agree

1. If diabetes is treated early, blindness due to diabetic retinopathy can be prevented.

1) Stongly disagree 2) Disagree 3) Don’t know 4) Agree 5) Strongly agree

1. Funduscopic examination should be done by ophthalmologist only.

1) Strongly disagree 2) Disagree 3) Don’t know 4) Agree 5) Stongly agree

1. Funduscopic examination by a non-ophthalmologist can help detect diabetic retinopathy.

1) Stongly disagree 2) Disagree 3) Don’t know 4) Agree 5) Strongly agree

1. Ophthalmology training in medical school adequately equips the GP to manage patients with eye complaints.
2. Strongly disagree 2) Disagree 3) Don’t know 4) Agree 5) Stongly agree

**Section 4 – Practice**

1. Do you test the vision of your diabetic patient ?

1) Yes 2) No

1. Do you examine the fundus (retina) of your diabetic patient ?

1) Yes 2) No

1. Do you refer diabetic patients for eye examination ?

1) Yes 2) No

1. Do you always have access to an ophthalmoscope at your workplace ?

1) Yes 2) No

1. Have you ever tried to do a fundus examination on your diabetic patient for the past six months?

1) Yes 2) No

6. Did you attend any seminar/training about DM and DR in the past year ?

1) Yes 2) No

1. What are the barriers during the implementation of DR screening at your workplace ?
